# Association of maternal nutritional status and small for gestational age neonates in peri-urban communities of Karachi, Pakistan: findings from the PRISMA study

**DOI:** 10.1186/s12884-024-06420-3

**Published:** 2024-03-22

**Authors:** Sobia Ambreen, Nida Yazdani, Abdul Salam Alvi, Muhammad Farrukh Qazi, Zahra Hoodbhoy

**Affiliations:** 1 Aga Khan University, Karachi, Pakistan; 2 Department of Pediatrics and Child Health, Aga Khan University, Karachi, Pakistan; 3 Ziauddin University, Karachi, Pakistan

**Keywords:** Maternal nutrition, Body mass index, Mid-upper arm circumference, Small-for-gestational age, Low birth weight

## Abstract

**Background:**

Early pregnancy nutritional status can be associated with adverse birth outcomes such as small-for-gestational age (SGA) and low birth weight (LBW). BMI (Body Mass Index) and MUAC (Mid-upper arm circumference) are easy to use assessments and are indicative of the pre-pregnancy nutritional status if obtained in the first trimester. This study primarily assesses the association of maternal nutritional status using BMI and MUAC with SGA in a community-based cohort of Pakistani women. It also aims to determine the predictive ability of MUAC and BMI in predicting SGA. Secondarily, we assessed the association between maternal nutrition and large for gestational age (LGA) and LBW.

**Methods:**

This study is a secondary analysis of an ongoing pregnancy cohort “Pregnancy Risk Infant Surveillance and Measurement Alliance (PRISMA)**“**in Ibrahim Hyderi and Rehri Goth, Karachi. PRISMA participants who were enrolled between January 2021 to August 2022 were included given they had a gestational age < 14 weeks confirmed via ultrasound, MUAC and BMI measurements were available and birth weight was captured within 72 hours. Multivariable logistic regression was used to determine an association between maternal nutritional status and SGA. The PRISMA study was approved by the Aga Khan University Ethics Review Committee (2021**–**5920-15**,**518).

**Results:**

Of 926 women included in the analysis, 26.6% (*n* = 247) had a low MUAC (< 23 cm) while 18.4% (*n* = 171) were underweight (BMI < 18.5 kg/m2). Nearly one third of low MUAC and underweight women delivered SGA infants (34.4 and 35.1% respectively). Underweight women and women with low MUAC had a statistically significant association with SGA (Underweight: OR 1.49, 95% CI 1.1,2.4; Low MUAC-OR 1.64, 95% CI 1.2,2.3) as well as LBW (Underweight: OR-1.63, 95% CI 1.1,2.4; Low MUAC-OR-1.63, 95% CI 1.2,2.3). ROC curves showed that MUAC and BMI had modest predictability for SGA (AUC < 0.7).

**Conclusion:**

Maternal nutritional status as indicated by BMI and MUAC are strongly associated with adverse pregnancy outcomes including SGA, LGA and LBW. Although MUAC and BMI are widely used to determine maternal nutritional status, they have poor predictive ability for newborn size. Further research is needed to identify other tools or a combination of tools to better predict adverse birth outcomes in resource-limited settings and plan interventions.

**Supplementary Information:**

The online version contains supplementary material available at 10.1186/s12884-024-06420-3.

## Introduction

Pre-conceptional nutritional status as well as maternal nutrition during pregnancy is pivotal to both mother and child’s health. Evidence suggests that maternal malnutrition can be associated with adverse birth outcomes such as preterm birth (PTB), low birth weight (LBW), small-for-gestational age (SGA) and intrauterine growth restriction (IUGR) [1–3]. The global prevalence of maternal underweight is reported to be 9.7% while in South Asia prevalence of maternal underweight status is nearly three times higher (24%) [4]. In Pakistan, a country grappling with poor maternal and neonatal indicators, women of reproductive age (WRA) face a triple burden of malnutrition: undernutrition, overweight/obesity, and micronutrient deficiencies all of which contribute to poor pregnancy outcomes [5]. According to the Pakistan National Nutrition Survey 2018, 14.4% WRA fall under the category of undernutrition defined as BMI < 18.5 kg/m2 [6].

Abnormal birth weight, whether classified small for gestational age (SGA) when birth weight falls below the 10th percentile or large for gestational age (LGA) when birth weight exceeds the 90th percentile for gestational age, are associated with increased infant mortality and morbidity [7, 8]. LGA infants often experience macrosomia, leading to complications during delivery, such as shoulder dystocia, Erb’s palsy, fractures, and neonatal asphyxia [9]. In contrast, SGA infants, particularly when born prematurely, are at a higher risk of various issues, including infections, hypothermia, hypoglycemia, respiratory problems, and feeding difficulties [9]. A recent Lancet Commission on Small Vulnerable Newborns (SVN) includes preterm, LBW and SGA infants as they collectively contribute significantly to poor neonatal outcomes [10, 11]. It is also notable that South Asia has the highest rates of SVN where 52.1% of all newborns are affected [11].

Maternal undernutrition is identified as a strong contributor to the development of SVNs [10]. Establishing nutritional status of the mother early in pregnancy is important so that adequate interventions can be proposed to prevent adverse pregnancy outcomes. Body Mass Index (BMI) and Mid-upper arm circumference (MUAC) are easy to use assessments for maternal nutritional status which are indicative of the pre-pregnancy nutritional status if obtained in the first trimester [12]. Several studies have highlighted MUAC as a viable alternative to BMI for nutritional screening of pregnant women in low-resource settings due to its ability to reduce chances of misclassification in case of late antenatal visits [13–15]. This study aims to contribute to the existing knowledge by assessing the association of maternal nutritional status using BMI and MUAC in predicting SGA in a community-based cohort of Pakistani women. It also aims to determine the predictive ability of MUAC and BMI in predicting SGA. The secondary objectives for this study included assessment of association of maternal nutritional status and LGA and LBW.

## Methods

### Study design and setting

The Pregnancy Risk Infant Surveillance and Measurement Alliance (PRISMA) is an ongoing prospective longitudinal cohort in Ibrahim Hyderi and Rehri Goth located in the peri-urban communities of Karachi, Pakistan. These communities are low socio-demographic communities situated in Bin Qasim town of the coastal region of Karachi. Pregnant women receive midwifery-led antenatal care services during their visit to the primary health care (PHC) facility where relevant laboratory and ultrasound investigations are performed, and women are facilitated for skilled delivery to higher level health care facilities. Further details regarding the PRISMA cohort have been described elsewhere [16].

### Study period and population

For this study, we included PRISMA participants who were enrolled between January 2021 to August 2022. Pregnant women with a viable intrauterine pregnancy < 14 weeks of gestation as confirmed by ultrasound, BMI and MUAC measurements in the first trimester and birth weights of their infant captured within 72 hours were included for this analysis. There was no predefined exclusion criteria for this analysis. All participants provided individual written informed consent before undergoing study procedures. The PRISMA study was approved by the Aga Khan University Ethics Review Committee (2021–5920-15,518).

### Study procedures

All women underwent ultrasound scans by certified sonologists at the time of enrolment to establish viable pregnancy and gestational age (GA). Trained community health workers (CHWs) conducted anthropometric measurements for women, including height (using SECA scale, model no. 213), weight (using SECA scale, model no. 876), and MUAC (using standardized UNICEF tapes), at each ANC visit. BMI was calculated using the WHO international BMI cut-offs and were categorized into underweight (BMI < 18.5 kg/m^2^), normal weight (18.5–24.9 kg/m^2^), overweight (25–29.9 kg/m^2^) and obese (≥30 kg/m^2)^ [17]. MUAC values were categorized as low MUAC for those with MUAC < 23 cm and normal if MUAC ≥23 cm [18]. These measurements were performed using SECA 876 weight machine, with a graduation of 100 g < 150 kg > 200 g and a maximum capacity of 250 kg. This underwent weekly calibration using 10 kg and 5 kg weights to ensure accurate and reliable measurements [19]. Additionally, height was measured using SECA 217 which covers a measuring range of 20–205 cm, while UNICEF MUAC tape were used (range from 1 to 59 cm) [20].

The pregnant mother was followed during pregnancy and for labor and delivery to capture relevant outcomes including baby’s birth weight within 72 hours of life. The primary outcome of this analysis was SGA while secondary outcomes included LGA and LBW. SGA and LGA were calculated based on INTERGROWTH 21 guidelines, utilizing the web-based INTERGROWTH 21 newborn size calculator [21, 22] while LBW was defined as birthweight less than 2500 g.

### Data analysis

The baseline characteristics of the study cohort such as current age, age at the time of marriage, were reported through mean and standard deviation using t-test and categorical variables such as education, wealth index, and other socio-demographic indicators as well as frequencies of outcome variables including SGA, LGA, LBW were reported as frequency and percentages using chi2 (Tables 1 and 2). Simple and multivariable logistic regression models were run for SGA, LGA and LBW separately to determine the association of these outcomes with BMI and MUAC while adjusting for age, age at marriage, smoking status, gravid status, comorbidities, severity of anemia and number of ANC visits in the current pregnancy. At the stage of crude logistic regression, a *p*-value of < 0.25 was taken for determining the level of significance and at multivariable level, *p*-value of < 0.05 was used for determining the level of significance. Stepwise model building approach was used for multivariable regression, however, the final adjusted models used were those that were not developed as stepwise rather those that adjusted all clinically important variables. Overall significance of the models were assessed to check fit of the models (*p* < 0.05) To assess the predictive capability of BMI and MUAC for SGA and LBW, we employed receiver operating curves (ROC) to calculate the area under curve (AUC). All statistical analyses were performed using STATA version 17.0.

**Table 1:** Sociodemographic, clinical, and obstetric characteristics of the participants included by status of BMI and MUAC

Variables	MUAC		BMI			
	Low MUAC *N* = 247 *n* (%)	Normal MUAC *N* = 679 *n* (%)	Underweight *N* = 171 *n* (%)	Normal *N* = 476 *n* (%)	Overweight *N* = 192 *n* (%)	Obese *N* = 87 *n* (%)
Woman’s current age^*^a^	26.1(5.6)	28.4(5.7)	27.1(6.1)	27.2(5.5)	28.6(5.6)	30.4(6.1)
Age at marriage*^a^	19.5(2.6)	19.9(2.9)	19.8(2.7)	19.5(2.8)	20.2(2.9)	20.1(3.1)
Education*						
No formal education	163(66.0)	409(60.2)	114(66.7)	308(64.7)	101(52.6)	49(56.3)
Primary or higher (Ref)	84(34.0)	270(39.8)	57(33.3)	168(35.3)	91(47.4)	38(43.7)
Wife’s occupation						
Unemployed (Ref)	144(58.3)	431(63.5)	111(64.9)	289(60.7)	119(62.0)	56(64.4)
Employed	103(41.7)	248(36.5)	60(35.1)	187(39.3)	73(38.0)	31(35.6)
Husband occupation						
Unemployed	3(1.2)	11(1.6)	1(0.6)	9(1.9)	4(2.1)	0
Employed (Ref)	668(98.4)	244(98.8)	170(99.4)	467(98.1)	188(97.9)	87(100)
No. of ANC visits						
1–3	16(6.5)	32(4.7)	9(5.3)	29(6.1)	7(3.6)	3(3.4)
> 4 (Ref)	231(93.5)	647(95.3)	162(94.7)	447(93.9)	185(96.4)	84(96.6)
Gravida^						
Primigravida (Ref)	54(21.9)	106(15.6)	40(23.4)	76(16.0)	31(16.1)	13(14.9)
Multigravida	193(78.1)	573(84.4)	131(76.6)	400(84.0)	161(83.9)	74(85.1)
Tobacco use^*						
Yes	95(38.5)	141(20.8)	82(48.0)	117(24.6)	27 (14.1)	10 (11.5)
No (Ref)	152(61.5)	538(79.2)	89(52.0)	359(75.4)	165(85.9)	77(88.5)
Anemia severity^b^^*						
No anemia (Ref)	82(33.2)	305(44.9)	57(33.3)	183(38.4)	96(50.0)	51(58.6)
Mild	121(49.0)	310(45.7)	83(48.5)	238(58.0)	78(40.6)	32(36.8)
Moderate to Severe	44(17.8)	64(9.4)	31(18.1)	55(11.6)	18(9.4)	4(4.6)
Co-morbidities*						
Yes	16(6.5)	69(10.2)	11(6.4)	35(7.4)	21(10.9)	18(20.7)
No (Ref)	231(93.5)	610(89.8)	160(93.6)	441(92.6)	171(89.1)	69(79.3)

^a^Presents mean (Standard deviation)

^b^Mild anemia (Hb < 11 & ≥9 mg/dl); moderate to severe anemia (Hb < 9 mg/dl)

^*P*-value< 0.05 MUAC vs independent variables

*P-value< 0.05 BMI vs independent variables

**Table 2:** Distribution of outcome variables by the status MUAC and BMI

Variables	MUAC		BMI			
	Low MUAC *N* = 247 *n* (%)	Normal MUAC *N* = 679 *n* (%)	Underweight *N* = 171 *n* (%)	Normal *N* = 476 *n* (%)	Overweight *N* = 192 *n* (%)	Obese *N* = 87 *n* (%)
SGA (<10TH centile) ^*						
** Yes**	85(34.4)	154(22.7)	60(35.1)	125(26.3)	37(19.3)	17(19.5)
** No**	162(65.6)	525(77.3)	111(64.9)	351(73.7)	155(80.7)	70(80.5)
**SGA Severity**						
No SGA (> = 10th centile & <90th centile)	162(65.5)	525(77.3)	111(64.9)	351(73.7)	155(80.7)	70(80.5)
Moderate (> = 3rd centile & <10th centile)	46(18.6)	85(12.5)	31(18.1)	72(15.1)	19(9.9)	9(10.3)
Severe (<3rd centile)	39(15.8)	69(10.2)	29(17.0)	53(11.1)	18(9.4)	8(9.2)
LGA (> = 90th centile) *						
** Yes**	6(2.4)	32(4.7)	5(2.9)	11(2.3)	14(7.3)	8(9.2)
** No**	241(97.6)	647(95.3)	166(97.1)	465(97.7)	178(92.7)	79(90.8)
LBW (< 2.5 kg) ^*						
** Yes**	78(31.6)	144(21.2)	55(32.2)	108(22.7)	38(19.8)	21(24.1)
** No**	169(68.4)	535(78.8)	116(67.8)	368(77.3)	154(80.2)	66(75.9)

^*p*-value < 0.05 MUAC vs independent variables

* *p*-value< 0.05 BMI vs independent variables

## Results

**Fig. 1:**
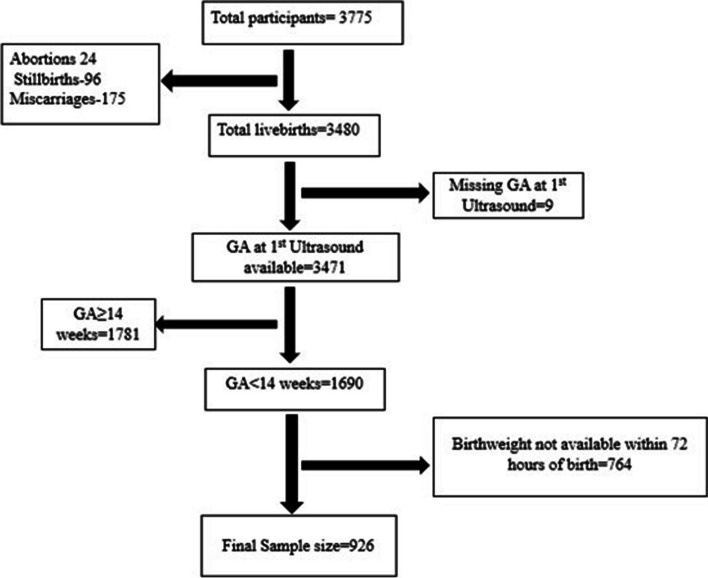
Flow diagram of all the participants included in the final analysis

The PRISMA Cohort enrolled 3775 participants from January 2021 to August 2022, of which 926 participants were included in this analysis (Fig. 1).

**Fig. 2:**
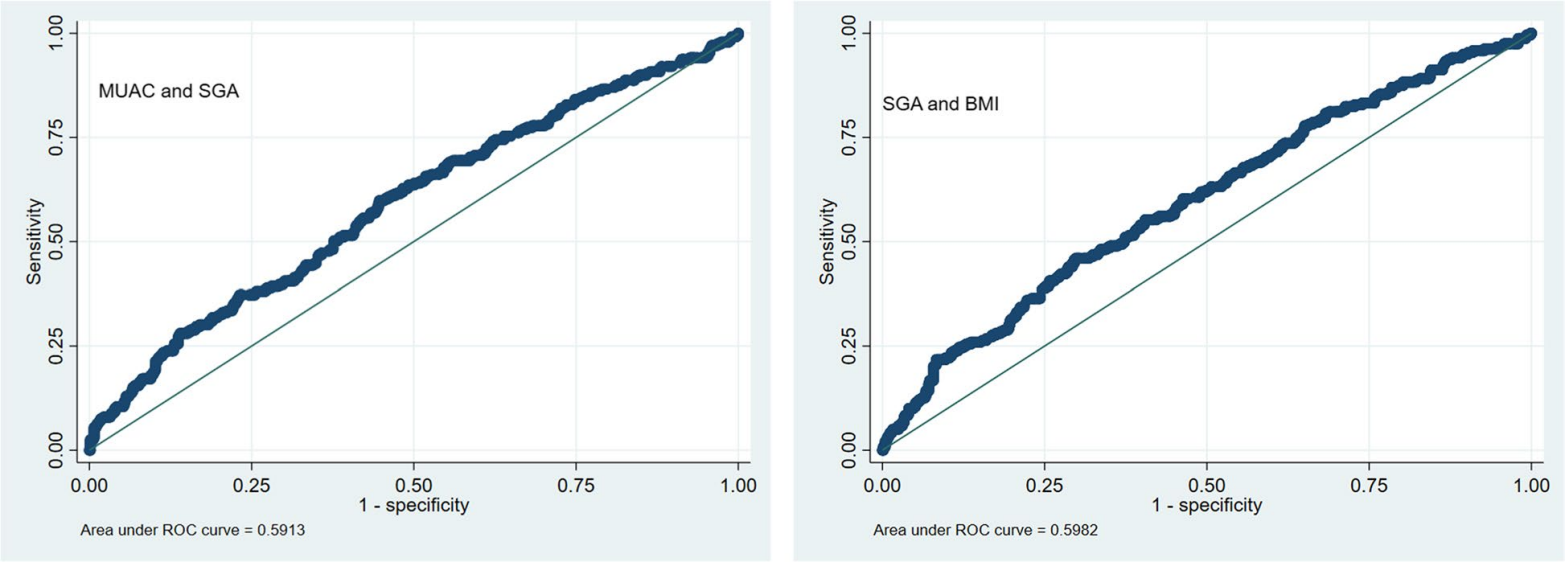
ROC curves for comparison of MUAC and BMI in predicting SGA

The study participants’ sociodemographic and clinical characteristics are presented in Table 1.

Among women with low MUAC, 66% (*n* = 163) women had no primary education, and 78.1% (*n* = 193) women were multigravida. Approximately 40% (*n* = 95) of women were smokers and 17.8% (*n* = 44) had moderate to severe anemia. Similarly, 48% (*n* = 82) of underweight women were smokers and 18.1% (*n* = 31) had moderate to severe anemia.

Among women with low MUAC, 34.4% (*n* = 85) women had neonates who were SGA and 21.2% (*n* = 144) were LBW. When stratified by BMI status, a similar burden of SGA neonates was reported (35.1%, *n* = 60) while the burden of LBW was higher (32.2%, *n* = 55) (Refer to Table 2).

When adjusted for woman’s current age, age at marriage, gravida, smoking status, comorbidities, severity of anemia and number of ANC visits, women with low MUAC had higher likelihood of SGA (*OR* = 1.64 95% CI 1.2, 2.3) compared to women with normal MUAC. Similarly, women who were underweight had higher likelihood of SGA (*OR* = 1.49,95% CI 1.1, 2.2) as compared to women with normal BMI (Table 3). Women with low MUAC and those who were underweight also had higher odds of LBW neonates (*OR* = 1.63, 95% CI 1.2, 2.3 and *OR* = 1.63, 95%CI 1.1, 2.4 respectively) (Table 3).

**Table 3:** Multivariable logistic regression models for birth outcomes (SGA, LGA and LBW)

	SGA aOR (95% CI)	LGA aOR (95% CI)	LBW aOR (95% CI)
**BMI**			
Underweight	1.49 (1.1, 2.2) *	1.38 (0.4, 4.1)	1.63 (1.1, 2.4) *
Overweight	0.70 (0.5, 1.1)	3.04 (1.3, 6.9) *	0.88 (0.5, 1.3)
Obese	0.72 (0.4, 1.3)	3.57(1.3, 9.6) *	1.14 (0.6, 2.0)
**MUAC**			
Malnourished	1.64 (1.2, 2.3) *	0.62 (0.2, 1.6)	1.63 (1.2, 2.3) *

*p-value< 0.05, aOR-Adjusted Odds Ratio

Both BMI and MUAC performed similarly and had a poor AUC of < 0.7 in predicting SGA. (Refer to Fig. 2) as well as LBW and LGA (refer to Figs. 1 and 2 in the supplement)

## Discussion

This study aimed to assess the association between maternal nutritional status in early pregnancy with adverse outcomes such as SGA, LGA and LBW in a community-based cohort in a low resource setting like Pakistan. Being underweight or having a low MUAC were significantly associated with SGA and LBW whereas the overweight and obese category of BMI were significantly associated with LGA, thus highlighting the impact of maternal nutritional status on neonatal size at birth.

Maternal nutrition and its impact on low-resource settings is multifaceted. Our study substantiates the exiting findings demonstrating a strong association between low MUAC and BMI as a risk factor for the likelihood of having SGA. This aligns with the global trends where underweight women face increased risks of fetal growth restriction (FGR), LBW and SGA [23–25]. A systematic review including LMICs reported that underweight women had 1.13 [95%CI, 1.01–1.27], 1.66 [95% CI, 1.50–1.84] and 1.85 [95%CI, 1.69–2.02] times higher odds of having a PTB, LBW and SGA, respectively. However, the absence of method used for specifying GA (LMP or Ultrasound) in this review may cause misclassification [26]. Additionally, a population-based prospective pregnancy registry from Pakistan and India linked underweight women to LBW, yet missed to include other outcomes like SGA or PTB [27]. Maternal pre-pregnancy overweight and obesity along with multiparity are strong determinants for delivering LGA infants [28]. A Swedish study reported that maternal overweight and obesity exhibited a significant association with LGA [28]. The current study enhances methodological rigor by employing first trimester ultrasounds for determining accurate GA, contributing evidence on association between maternal nutritional status and poor pregnancy outcomes like SGA, LGA and LBW.

BMI has conventionally served as a gauge for maternal nutritional status and fetal growth prediction [29]. However, growing evidence is suggesting that MUAC could be used as an alternative in assessing pregnancy outcomes [15, 30]. A study in India found a significant association (aOR = 7.91, *p*-value < 0.001) and also a moderate positive correlation between MUAC and BMI (r = 0.57, *p* < 0.001), suggesting MUAC to be a simple yet effective tool for assessing maternal nutritional status in pregnancy especially in low resource settings [30]. Results from a Zambian study assessing BMI and MUAC for predicting SGA revealed an inadequate overall predictive model (AUC_BMI_ = 0.66, AUC_MUAC_ = 0.68). However, it showed high discriminatory power for severe SGA in HIV-positive women (AUC_BMI_ = 0.8 and AUC_MUAC_ = 0.83) [13]. Our study also showed a slightly higher odds of SGA (*OR* = 1.64) with MUAC as compared with BMI (*OR* = 1.49); however, the predictive ability of both BMI and MUAC was poor on ROC curves.

This study had a few strengths and limitations. We confirmed the gestational age of each pregnant woman through ultrasound in early pregnancy which gave us accurate dating of the pregnancies. This is a major strength of this study as gestational age based on ultrasound is more accurate than LMP in determining gestational age [31, 32]. BMI and MUAC were measured with standard instruments and trained staff in a prospective manner. However, there are certain limitations to this study. Birthweight was not available for 21% of the women enrolled in the PRISMA study which could have led to selection bias. Also, the sample for this study was obtained from communities in the coastal regions of Karachi where the majority have a lower socioeconomic background thus may not be a representative sample for the country. Furthermore, we were unable to analyze gestational weight gain over the course of pregnancy due to insufficient data.

## Conclusion

This study concludes that early maternal nutritional status determined by BMI and MUAC is associated with adverse pregnancy outcomes including SGA,LGA, and LBW in low resource settings. BMI and MUAC are the most common and easily available tools to assess the mother’s nutritional status in early pregnancy despite its limited performance. Considering the importance of nutritional assessment of the mother in early pregnancy, there is a need to explore other proxy measures such as dietary diversity score, mid-arm muscle area or use combination of existing tools to enhance our understanding of this important association. These measures can then be effectively utilized in resource limited settings to assess the effect of nutritional interventions in pregnancy and their impact on neonatal outcomes.

### Supplementary Information

**Supplementary Material 1.**

## Data Availability

The dataset and material related to this study can be available from the corresponding author on reasonable request.

## Abbreviations

GA: Gestational age
BMI: Body Mass Index
MUAC: Mid upper arm circumference
SGA: Small-for-gestational age
LGA: Large-for-gestational age
LBW: Low birthweight
SVN: Small vulnerable newborns
FGR: Fetal growth restriction
IUGR: Intra-uterine growth restriction
WHO: World Health Organization
PRISMA: Pregnancy Risk Infant Surveillance and Measurement Alliance
ROC: Receiver operating curve
AUC: Area under curve
LMICs: Low- and middle-income countries
