# Supplementary material for: Association of maternal nutritional status and small for gestational age neonates in peri-urban communities of Karachi, Pakistan: findings from the PRISMA study
Source: BMC Pregnancy Childbirth. 2024 Mar 22;24:214. doi: 10.1186/s12884-024-06420-3 (PMC10958913; doi:10.1186/s12884-024-06420-3)
Supplement: Supplementary file 1 — Supplementary Material 1. [file 12884_2024_6420_MOESM1_ESM.docx]

**SUPPLEMENTARY MATERIAL**

**Figure 1: ROC curves for LBW vs BMI and LBW vs MUAC**

| 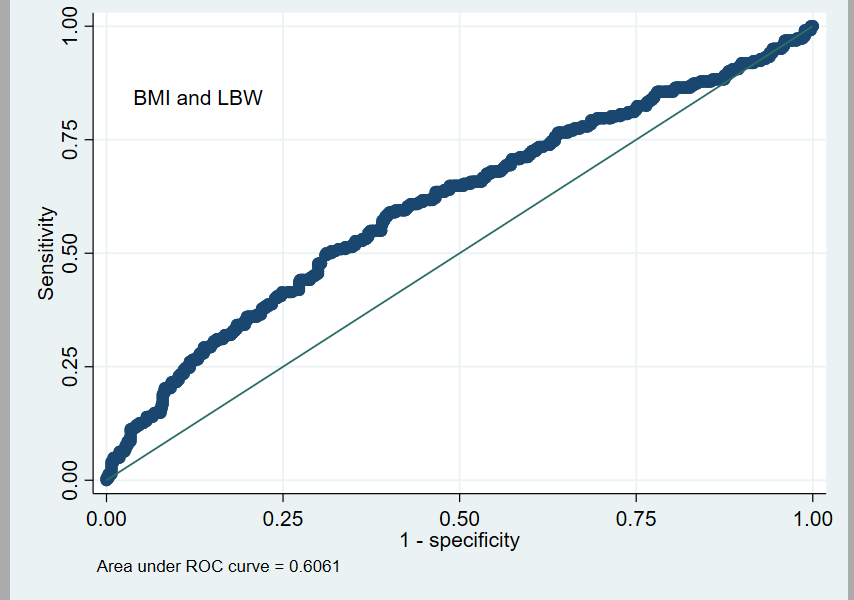 | 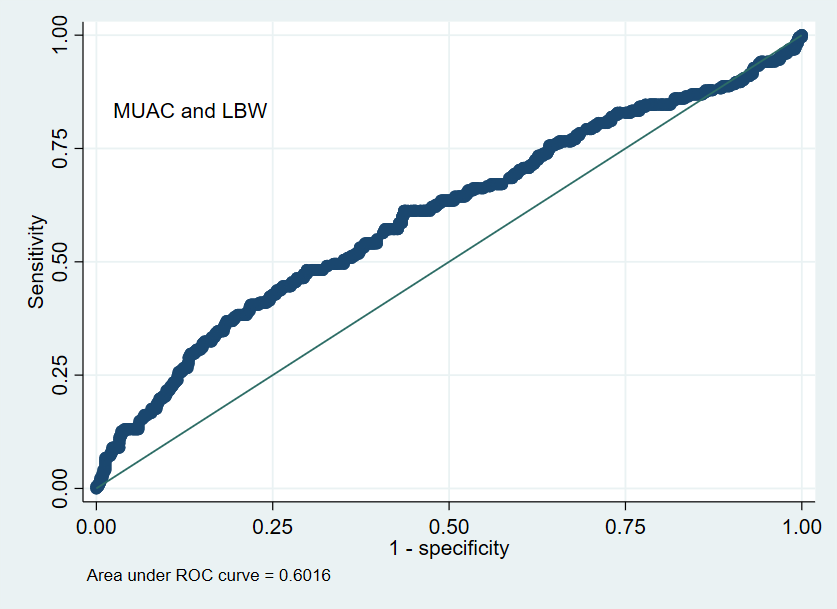 |
| --- | --- |

**Fig 2: ROC curves for BMI vs LGA and MUAC vs LGA**

| 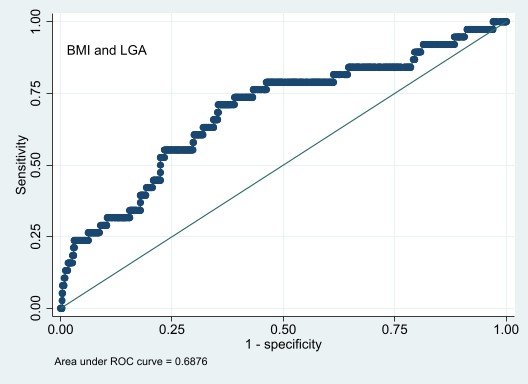 | 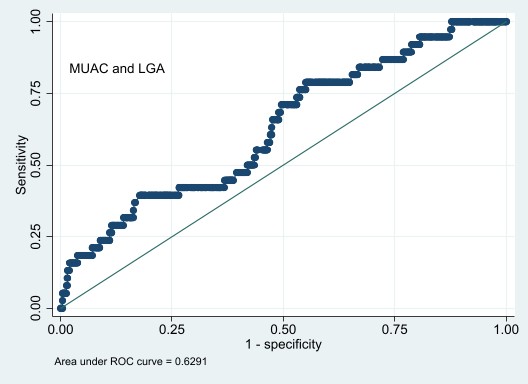 |
| --- | --- |
